# Calyx and dimorphic neurons of mouse Scarpa's ganglion express histamine H3 receptors

**DOI:** 10.1186/1471-2202-10-70

**Published:** 2009-06-29

**Authors:** Simona Tritto, Laura Botta, Valeria Zampini, Gianpiero Zucca, Paolo Valli, Sergio Masetto

**Affiliations:** 1 Dipartimento di Fisiologia, Università degli Studi di Pavia, 27100, Italy

## Abstract

**Background:**

Histamine-related drugs are commonly used in the treatment of vertigo and related vestibular disorders. The site of action of these drugs however has not been elucidated yet. Recent works on amphibians showed that histamine H3 receptor antagonists, e.g. betahistine, inhibit the afferent discharge recorded from the vestibular nerve. To assess the expression of H3 histamine receptors in vestibular neurons, we performed mRNA RT-PCR and immunofluorescence experiments in mouse Scarpa's ganglia.

**Results:**

RT-PCR analysis showed the presence of H3 receptor mRNA in mouse ganglia tissue. H3 protein expression was found in vestibular neurons characterized by large and roundish soma, which labeled for calretinin and calbindin.

**Conclusion:**

The present results are consistent with calyx and dimorphic, but not bouton, afferent vestibular neurons expressing H3 receptors. This study provides a molecular substrate for the effects of histamine-related antivertigo drugs acting on (or binding to) H3 receptors, and suggest a potential target for the treatment of vestibular disorders of peripheral origin.

## Background

The vestibular, or balance, organs inform the brain about the movements and position of the head. The sensory receptors of the vestibular apparatus are the hair cells. Two types of hair cells are present in mammalian vestibular organs, called Type I and Type II hair cells, which differ substantially in their afferent contact. The Type I hair cell's basolateral membrane is completely enveloped in a large afferent nervous terminal, called a calyx. In contrast, Type II hair cells are contacted by numerous bouton-like afferent terminals. The soma of afferent neurons are located in the Scarpa's ganglion. Central projections of the vestibular nerve contact secondary neurons located in the brainstem (vestibular nuclei) and in the cerebellum, which provide the anatomical basis for the vestibular reflexes.

Most cases of dizziness and vertigo are due to problems in vestibular organs, presumably resulting in wrong afferent signaling interpreted by the brain as if the head is moving or turning when it is actually not.

Among the pharmacological compounds commonly used in the symptomatic treatment of vertigo and related vestibular disorders are histamine-related drugs, such as promethazine [1], dimenhydrinate [2] and cinnarizine [3], assumed to act at H1 histamine receptors, and betahistine [4,5], which has been shown to act as an H3 histamine receptor antagonist [6]. However, the site/s of action of these drugs have not been defined. Histamine H1, H2 and H3 receptor subtype expression has been reported in rat vestibular nuclei [7,8], suggesting the possibility that the antivertigo effect of histamine-related drugs occurs centrally. On the other hand, experiments performed on amphibian semicircular canals have shown that H1 and H3 receptor ligands affect the sensory input recorded from the afferent vestibular neurons [9-11], indicating a possible peripheral site of action for these drugs. Consonant with the latter studies, we recently reported that H1 receptors are expressed in frog and mouse hair cells [12].

In the present study we report that a subpopulation of mouse Scarpa's ganglion neurons innervating both Type I and Type II hair cells express H3 receptors.

## Methods

Experiments were performed on mice (Swiss CD-1) about 3 weeks old. All experimental procedures involving animals were approved by the Ministero Italiano della Sanità, and comply with the European international laws on animal research. Prior to any surgery, deep and irreversible anesthesia was obtained by means of alothane.

### RT-PCR

For RT-PCR experiments, brain and vestibular ganglia were removed immediately after decapitation of the animal. The superior and inferior vestibular ganglia were removed together. Since both ganglia similarly stained for the H3 receptor (H3R) antibody, no differential analysis was performed.

Total RNA was extracted from mouse brain (1 mouse/experiment for 3 experiments) and Scarpa's vestibular ganglia (50–100 mice/experiment for 3 experiments) using QIAzol Lysis Reagent (QUIAGEN, Italy). Single-strand cDNA was synthesized from RNA (1 μg) using random hexamers and M-MLV Reverse Transcriptase (Invitrogen, USA). Subsequently, PCR (30 s at 96°C, then 30 s at 62°C or 69°C for β-actin and H3 respectively, followed by 30 s at 72°C for 33 cycles) was performed on 2.5 μl cDNA using specific primers for H3 receptors designed in accordance with the published sequence. H3 receptors display alternative splicing mainly in the region of the third intracellular loop [13]. Primers for H3 receptors were designed around a nonspliced region, so that all variants would be detected, although not differentiated. The forward and reverse sequences were: 5'-TTCAACATCGTGCTGATCAG-3' 5'-TGTTCAGGTAGATGCTGAGG-3. As an internal control for cDNA yield, parallel RT-PCR was performed using specific primers for mouse β-actin. The primer sequences were: 5'-CAGATCATGTTTGAGACCTT-3' 5'-CGGATGTCMACGTCACACTT-3'. Negative control experiments were always performed by omitting the reverse transcriptase. The molecular weight (MW) of PCR products was estimated using the DNA MW marker VIII (Roche Molecular Biochemicals, Italy).

### Immunofluorescence

For immunofluorescence experiments ganglia were dissected from mice (n = 8), embedded in Jung Tissue Freezing Medium (Leica Microsystems, Italy) and immediately frozen in liquid nitrogen. Multiple 10 μm cryostat sections were obtained from the frozen samples, washed with phosphate-buffered saline (PBS) solution, blocked for 60 min with bovine serum albumin (BSA) 3%, rinsed with PBS and then incubated for 2 hours with rabbit anti-rat H3R IgG (Alpha Diagnostic International, USA), diluted 1:50 in PBS. After a rinse in PBS (15 min), the sections treated with anti-H3 were incubated (30 min) with Alexa-fluor 488-conjugated anti-rabbit IgG (1:1000) (Invitrogen, Italy).

No immunostaining was observed in control sections incubated with H3R antibodies preadsorbed with 20 M excess of the relative control peptide (Alpha Diagnostic International, USA), thus confirming anti-H3R antibody specificity.

For the colocalization study of histamine receptors and calretinin or calbindin, slices were incubated with rabbit anti-rat H3R IgG (Alpha Diagnostic International, USA) plus goat calretinin antiserum (Alpha Diagnostic International, USA), or plus goat calbindin antiserum (D28k-c20; Santa Cruz Biotechnology Inc., Germany), all diluted 1:50 in PBS except for calretinin which was diluted 1:250. After a rinse in PBS (15 min) the sections were incubated with Alexa Fluor 488-conjugated anti-rabbit IgG (1:1000) and Alexa Fluor 546-conjugated anti-goat IgG (1:1000). The immunostained slices were examined using the AFTER^® ^fluorescence LED module (Fraen Corporation, Italy). Nuclei were counterstained with DAPI (ProLong^® ^Gold antifade reagent with DAPI, Invitrogen, Italy). Slides were mounted on an Olympus BX41 light microscope, and digital images were captured with an Olympus Camedia C-5050 zoom digital camera (Olympus Italia, Italy). Several images were also acquired by mean of a TCS SP2 LEICA confocal microscopy system equipped with a LEICA DM IRBE inverted microscope.

In two additional experiments ganglia were not dissected directly from the mouse, but the petrous bone containing the whole inner ear was dissected out surgically and post-fixed in 4% paraformaldehyde in 0.1 M PB for 2–3 hr. Bone was decalcified for at least 1 week in a 120 mM EDTA solution at 4°C before further processing into paraffin. Serial paraffin sections (10 μm) were brought to water, washed with phosphate-buffered saline (PBS) solution, blocked for 60 min with BSA 3%, rinsed with PBS and then incubated for 2 hr with anti-H3R antibody (Alpha Diagnostic International, USA) diluted 1:50 in PBS. After a rinse in PBS (15'), the slices were treated with anti-H3R and incubated (30') with Alexa-fluor 488 anti-rabbit IgG (Invitrogen, Milan, Italy). Slices were then washed 3 × 5 min with PBS, mounted in ProLong^® ^Gold antifade reagent with DAPI (Molecular Probes) and examined with a TCS SP2 LEICA confocal microscopy system equipped with a LEICA DM IRBE inverted microscope.

### Neuron count and size

To estimate the percentage of ganglia neurons expressing H3 receptors, we calculated the ratio of cells staining for both H3 antibody and DAPI vs. cells staining for DAPI only in ganglia slices. All nuclei that were clearly recognizable were counted in slices. Since slices were 10 μm thick, and neuron soma ranged between 10 and 30 μm, in serial sections a same neuron could be counted twice. This would have yielded to an overestimation of H3 expressing neurons as they showed a larger soma (see Result sections). Therefore, we skipped one slice every count. The same was done for calculating the percentage of calretinin-positive neurons.

Finally, in some experiments ganglia tissue was stained with toluidine blue or hematoxylin/eosin, which stain neuron nuclei and cytoplasm. The diameter of each nucleus and soma was obtained by averaging the largest and the shortest diameters measured.

Analysis was performed with Microcal Origin (Version 6.0. Microcal Software, Northampton, MA, USA) and Microsoft Excel V. 5 (Microsoft, Redmond, WA, USA). Data are presented as means ± standard deviation; n = number of cases.

## Results

### Expression of H3 histamine receptor mRNA in the mouse vestibular ganglia tissue

**Figure 1:**
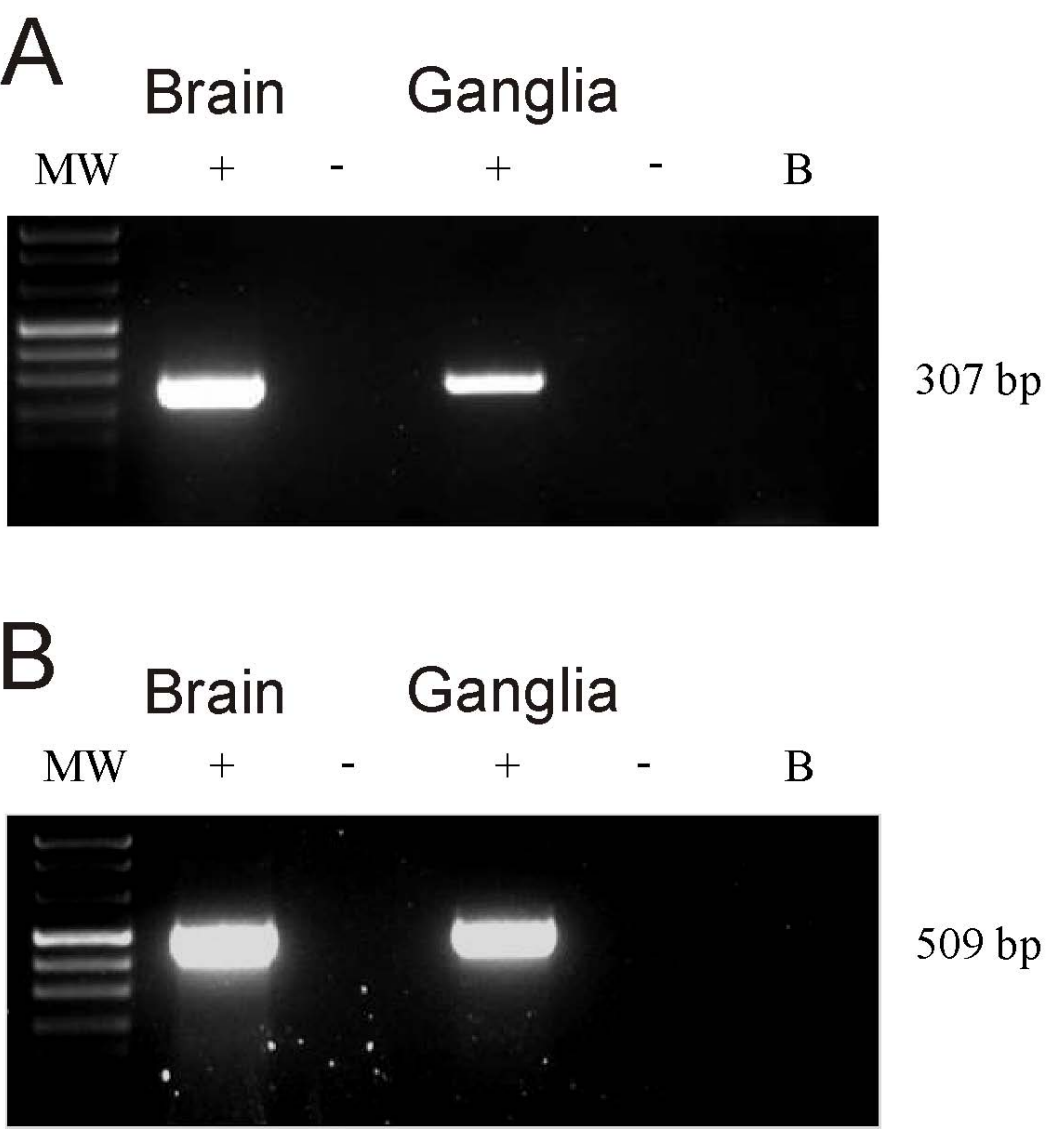
**Reverse transcriptase-PCR analysis in the mouse vestibular ganglia**. **A: **mRNA RT-PCR for H3 receptor expression in mouse brain and Scarpa's ganglia. B: mRNA RT-PCR for β-actin expression in mouse brain and Scarpa's ganglia. The 307 and 509 bp bands correspond to the H3 and β-actin specific PCR products, respectively. β-actin mRNA was used as the internal control.

RT-PCR was performed to evaluate the expression of H3 receptor mRNA in mouse vestibular ganglia. Fig. 1A shows representative agarose gel electrophoresis results for H3 mRNA expression in mouse vestibular ganglia and brain. Bands for H3 mRNAs were obtained for both control tissue (brain) and ganglia. The good quality of the mRNA extracted from the labyrinth was confirmed by the presence of the β-actin band (Fig. 1B).

### H3 protein receptor immunofluorescence

Immunofluorescence experiments were performed both in slices obtained from ganglia dissected and fixed, and following decalcification of the whole petrous bone (see Methods). No obvious differences were found between the two methods.

**Figure 2:**
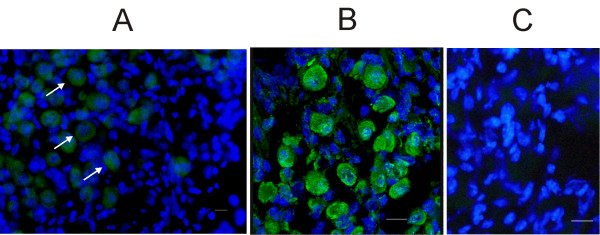
**Immunofluorescence of histamine H3 receptors in mouse Scarpa's ganglion**. A: light microscope photomicrograph of a Scarpa's ganglion slice. H3 receptors appeared expressed in neurons characterized by large nuclei. White arrows point at three such neurons. B: confocal light photomicrograph of an enlarged image from a different ganglion slice. C: light microscope photomicrograph from a different ganglion slice, following H3R antibody preabsorption (see Methods). No labeling for H3R is detectable. Green: H3R antibody; Blue: DAPI. Slice thickness: 10 μm. Calibration bar: 20 μm.

On average, 30% of all neurons in the slices (± 9%; n = 1264 – tissue from 8 ganglia) stained for the H3R antibody. This neuronal subpopulation was characterized by a large and roundish nucleus and soma, and by an unevenly dense distribution of chromatin in the nucleoplasm (Fig. 2A and 2B). The specificity of the H3R antibody was confirmed by preabsorption experiments with the antibody control peptide (Fig. 2C).

Large soma neurons in rodent vestibular ganglia were reported to stain for calretinin and calbindin, two calcium binding proteins, whereas small soma neurons stained for peripherin [14,15]. We therefore investigated if H3R-positive neurons fitted in the calretinin/calbindin neuronal subpopulations.

### Co-labeling of H3 receptors and calretinin/calbindin

**Figure 3:**
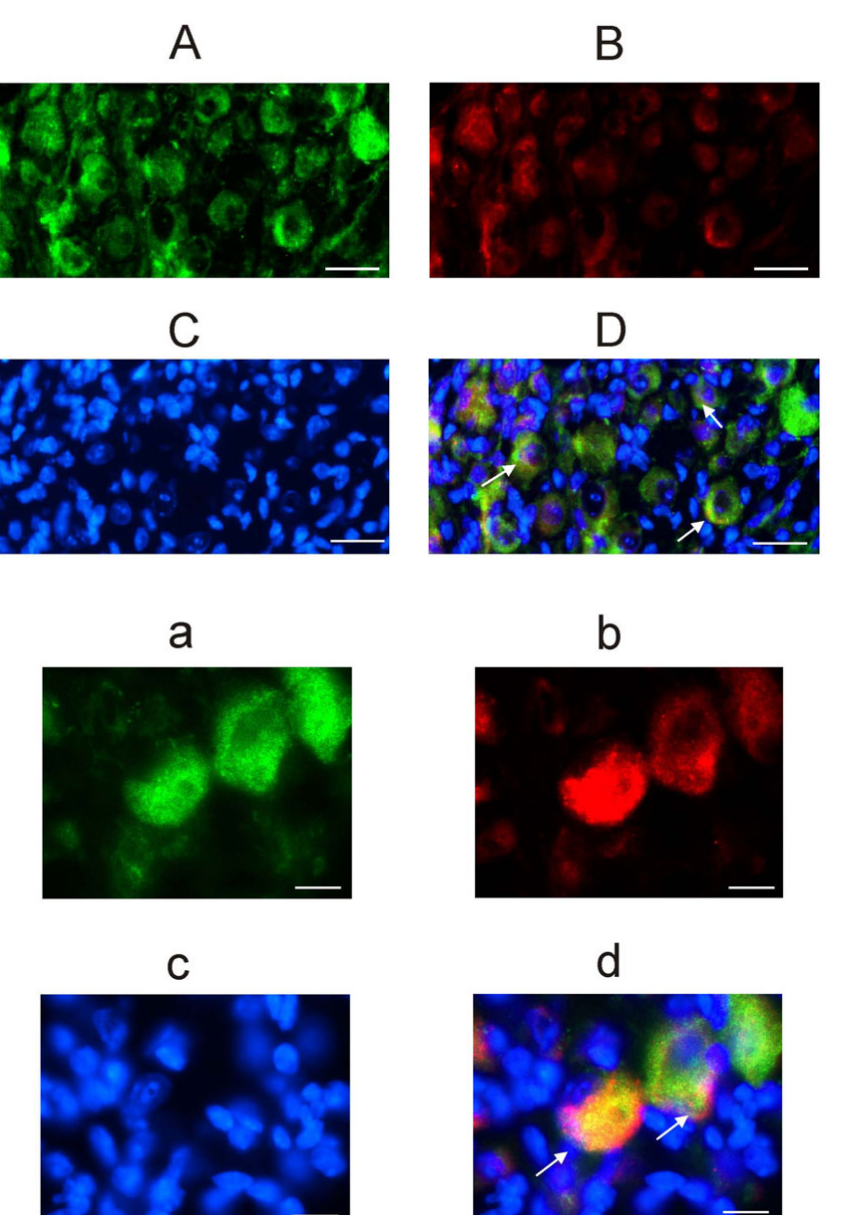
**Immunofluorescence of calretinin and histamine H3 receptor in mouse Scarpa's ganglion**. A: confocal image of a Scarpa's ganglion slice immunolabeled for H3 receptors. B: Immunolabeling for calretinin, same field as in A. C: nuclei counterstained with DAPI, same field as in A and B. D: overlapped images; yellow indicates the colocalization of calretinin and H3 receptors (three examples shown by arrows). Calibration bar: 10 μm. Cryostat slices thickness: 5 μm. Green: H3R Alexa Fluor 488; Red: Calretinin Alexa Fluor 546; Blue: DAPI. a, b, c and d are larger magnifications from a different slice, same antibodies and description as above. Calibration bar: 20 μm.

**Figure 4:**
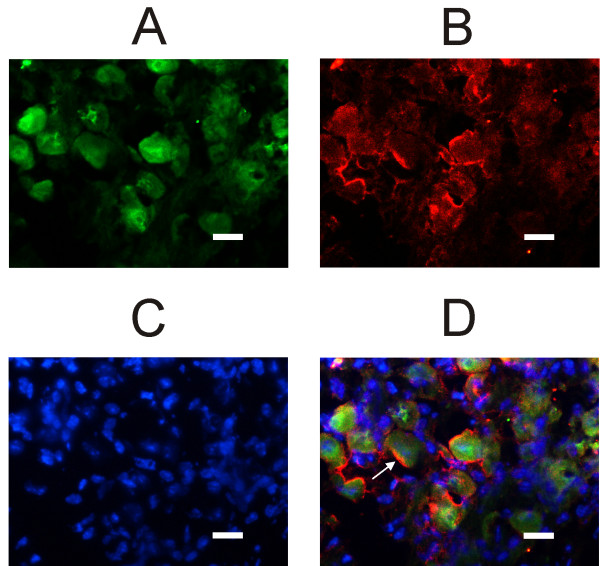
**Immunofluorescence of calbindin and histamine H3 receptor in mouse Scarpa's ganglion**. All confocal images refer to the same field. A: slice immunolabeled for H3 receptors. B: Immunolabeling for calbindin. Note that, although staining is less homogeneous than in A, there is a substantial overlap of the stained soma. C: nuclei counterstained with DAPI. D: overlapped images; yellow indicates the co-localization of calbindin and H3R. Calibration bar: 20 μm. Cryostat slices thickness: 5 μm. Green: H3R Alexa Fluor 488; Red: Calbindin Alexa Fluor 546; Blue: DAPI.

Fig. 3 shows that a subpopulation of H3R-positive neurons additionally labels for calretinin, which is considered a pure marker of vestibular calyx afferents [14,15]. The co-localization with calretinin was observed in 21% (± 4.9%; n = 1264 – tissue from 8 ganglia) of H3R positive neurons. The large soma neurons labeling for H3R but not calretinin should include dimorphic neurons innervating both Type I and Type II hair cells. Calyx and dimorphic neurons were reported to immunostain for calbindin [14,15]. To confirm this assumption, in six slices from two different ganglia we tested if H3 receptors co-localized with calbindin. As shown in Fig. 4, practically all H3R-positive neurons also stained for calbindin.

### Size of H3-positive neurons

**Figure 5:**
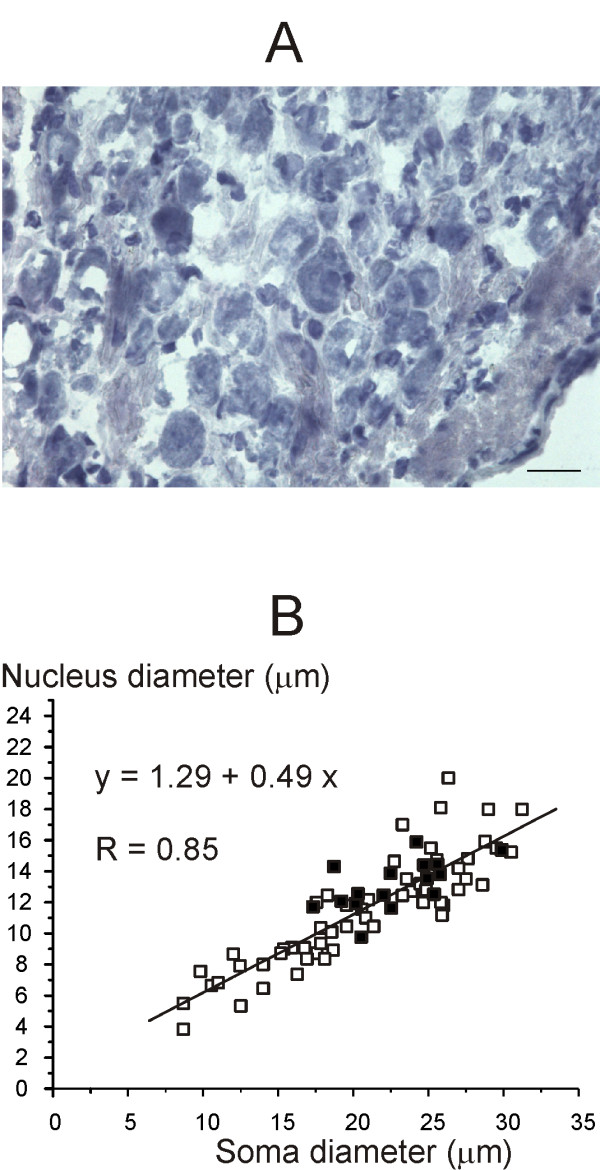
**Scarpa's ganglion neurons size**. A: Photomicrograph of a typical ganglion slice counterstained with ematoxylin/eosin, which stains both nucleus and cytoplasm. Calibration bar: 20 μm. B: scatter plot showing neuronal soma and relative nucleus diameter in a typical slices stained with hematoxylin/eosin (squares) and in a typical slice stained for the H3R antibody (filled squares). The equation for the regression line and the correlation coefficient value are shown in figure.

Antibodies for calretinin, calbindin and H3 receptors apparently marked only neurons characterized by large soma and nucleus. However, since DAPI only stains the nucleus, it is not possible to exclude that some neurons not expressing H3 receptors or calretinin/calbindin had a small nucleus but a large soma. To test for this possibility we measured the soma and nucleus of neurons counterstained with hematoxylin/eosin or toluidine blue, which stain both nucleus and cytoplasm (see e.g. Fig. 5A). As depicted in Fig. 5B, soma and nucleus diameter of neurons covary (correlation coefficient: 0.85), suggesting that neurons showing a small nucleus also have a small soma. Furthermore, H3-positive cells appeared grouped in the upper part of the graph distribution, corresponding to neurons characterized by largest soma and nucleus. The mean nucleus diameter was 16.4 μm (± 4.2; n = 382) for H3 positive neurons and 16.9 μm (± 13.7; n = 223) for calretinin-positive neurons respectively.

## Discussion

Our RT-PCR experiments showed that mouse vestibular ganglia express the mRNA for H3 histamine receptors. Moreover, antibodies anti-H3R protein stained neurons characterized by a relatively large soma and nucleus. This is the first report of H3 receptors expression by primary vestibular neurons, suggesting they could represent a peripheral target of histamine-related drugs, besides histamine receptors expressed by neurons of the vestibular nuclei, which are a central target.

About one fifth (21%) of H3R-positive neurons expressed calretinin, and virtually all co-labeled for calbindin. Calretinin was reported to selectively mark pure vestibular calyx afferents which innervate Type I hair cells only, while calbindin additionally labeled dimorphic afferents contacting both Type I hair and Type II hair cells [14,15]. A third neuronal subpopulation was also identified, characterized by the smallest soma, which stained with peripherin; these neurons make bouton-like synapses on Type II hair cells only.

Taken together, our results indicate that H3 receptors are expressed by calyx and dimorphic, but not bouton, afferent neurons.

Calyx, dimorphic and bouton afferents, beside innervating different proportions of Type I vs. Type II hair cells, show distinct regional distributions in the sensory epithelia [15,16] and diverse resting and dynamic discharge properties [17]. The reason for this afferent diversity is not fully understood, although it has been proposed that it might serve to match different vestibular reflexes [17]. It seems interesting here to note that the expression of H3 receptors represents an additional difference among primary vestibular afferent neurons.

### Role of H3 receptors in vestibular ganglion neurons

H3 antagonists were shown to inhibit the afferent discharge recorded from the vestibular nerve of the axolotl [10,11]. It was proposed that H3 receptors were localized post-synaptically to the hair cells, i.e. at the afferent vestibular nerve, since H3 antagonists were still effective when the synapse was blocked by a high Mg^2+^/low Ca^2+ ^solution [11]. This hypothesis is consistent with the present results, and also with a recent study showing no evidence for H3 receptors expression by vestibular hair cells [12].

On the whole, these results suggests that histamine, and related compounds, can affect the excitability of primary vestibular neurons through direct action on H3 receptors. This conclusion, however, does not exclude a possible pre-synaptic effect of H3 receptors upon glutamatergic neurotransmission between Scarpa's ganglion neurons and second order neurons located in the vestibular nuclei and in the vestibolocerebellum. Although in fact H3 receptors are commonly found on histaminergic nerve endings where, by acting as autoreceptors, they are responsible for histamine inhibiting its own release, they have also been found on nonhistaminergic endings whereby histamine can inhibit release of different neurotransmitters, among which glutamate [18].

## Conclusion

The main original finding of the present study is that mouse vestibular ganglion neurons express histamine H3 receptors. Interestingly, data are consistent with H3 receptors being expressed by calyx and dimorphic, but not bouton, afferents. This suggests the possibility that histamine modulates distinct sensory pathways. Finally, it is possible to speculate that the anti-vertigo action reported for some histamine-related drugs involves H3 receptors expressed by primary vestibular neurons.

## Authors' contributions

TS carried out the molecular biology and the immunofluorescence experiments, and participated in the design of the experiments and analysis of data. BL participated in the molecular biology and immunofluorescence experiments. ZV performed the surgery and participated in the immunofluorescence study. ZG participated in the design of the study, analysis of the results and critical revision. VP participated in the design of the study and helped to draft the manuscript. MS conceived of the study, coordinated the participants and wrote the final manuscript. All authors read and approved the final manuscript.
